# Quality of breaking bad news to patients diagnosed with neoplasia of the uterine cervix

**DOI:** 10.1007/s00432-023-05442-2

**Published:** 2023-10-04

**Authors:** Pia von Blanckenburg, Christhardt Köhler, Anja Petzel, Anne Jülicher, Viola Schneider, Achim Schneider

**Affiliations:** 1 Department of Clinical Psychology and Psychotherapy, Philipps-University of Marburg, Gutenbergstr. 18, 35032 Marburg, Germany; 2 Institute for Cytology and Dysplasia, Hohenzollerndamm 123, 14199 Berlin, Germany

**Keywords:** Bad news, SPIKES protocol, Cervical cancer, Communication

## Abstract

**Objective:**

Little is known about the quality of receiving bad news (BN) for women diagnosed with cervical neoplasia. We evaluated adherence to the SPIKES protocol in three cohorts of women with different stages of the disease and treatment modalities.

**Patients and methods:**

We included women with cervical cancer who underwent radical vaginal trachelectomy (RVT group, *n* = 110), radical hysterectomy or chemo-radiation (HE/RCT group, *n* = 101), and women with CIN 3 treated by loop excision (CIN group, *n* = 108). We asked the participants about how they received the bad news delivery in reality and how they would envision an ideal communication process based on the main items of the SPIKES protocol. The participants filled out a questionnaire with 38 items of the Marburg Breaking Bad News (MABBAN) Scale representing the six SPIKES subscales.

**Results:**

Only 72% of all patients reported being satisfied with their BBN experience. The following factors were considered important by 90% of the patients: an undisturbed atmosphere, taking enough time, coherent explanation of the disease, and the possibility to ask questions. However, the reality of their experiences fell significantly short of their expectations. Asking about the patient’s knowledge of the disease, addressing their concerns, allowing them to show emotions, providing clarity about the change in quality of life, informing them about alternative therapies, and involving them in further planning were also significantly lacking in the actual BBN encounters compared to the patients’ preferences. The experience of RVT patients was more negative compared to the HE/RCT patients (*p* = 0.036). The CIN patients had an overall satisfactory impression (*p* < 0.0001).

**Conclusion:**

The process of breaking bad news in German women diagnosed with cervical neoplasia requires substantial improvement. The SPIKES protocol can be used as a guideline for enhancement but should be supplemented by incorporating a second consultation as the norm rather than the exception. Continuous monitoring and improvement of the quality of BBN is recommended for all oncologic institutions, utilizing the MABBAN questionnaire as a valuable tool.

**Supplementary Information:**

The online version contains supplementary material available at 10.1007/s00432-023-05442-2.

## Introduction

The comment, “You see only the disease, but not the human being involved,” highlights the perspective of a patient diagnosed with cervical cancer who sought a second opinion from A.S. several years ago regarding further therapeutic steps. Dissatisfied with the communication of her life-threatening diagnosis and treatment, this patient perceived the delivery of bad news (BN) as suboptimal. Bad news can be defined as any information that adversely and significantly affects an individual’s perception of their future (Buckman 1992). However, accurately estimating the impact of bad news requires evaluating the recipient’s expectations and understanding.

Breaking bad news (BBN) is a complex communication task that extends beyond verbal delivery (Parker et al. 2001). It necessitates addressing patients’ emotions, fears, and psychological status, involving them in decision-making without causing undue stress or frustration, managing their expectations for a cure, considering the involvement of loved ones, family members, and friends, and navigating the challenge of maintaining hope in challenging circumstances (Baile et al. 2000). To train physicians in effectively delivering bad news, a six-step protocol known as SPIKES was developed by a panel of experts (Table 1). This protocol, evaluated by 500 participants at a symposium in ASCO 1998, emphasizes setting up the interview in a suitable environment, assessing the patient’s perception and understanding, determining the patient’s desired level of information, providing knowledge and information in an open, honest, and optimistic manner while avoiding medical jargon, addressing the patient’s emotions with empathy, and discussing strategies and next steps (Baile et al. 2000).

**Table 1:** Steps of the SPIKES protocol

STEP 1: SETTING UP the interview
STEP 2: Assessing the patient’s PERCEPTION
STEP 3: Obtaining the patient’s INVITATION
STEP 4: Giving KNOWLEDGE and information to the patient
STEP 5: Addressing the patient’s EMOTIONS with empathic responses
STEP 6: STRATEGY and SUMMARY

The evaluation of BBN in women with gynecologic cancer remains limited, with few studies available. An English survey of 359 patients diagnosed with epithelial ovarian cancer over a 2 year period revealed poor quality of BBN, including inadequate recording of prognosis (21%) and limited collusion with relatives (10%), which was more common in patients older than 65 years. Furthermore, information was frequently poorly documented in patient records (Kirwan et al. 2003). A review conducted in England focused on women diagnosed with cervical cancer detected through screening programs. The review concluded that while patients experience distress upon disclosure, there are also potential positive aspects such as enhanced trust and improved perception of care. Recommendations included the availability of patient representatives or psychologists and the value of input from pathologists (Sherman et al. 2013).

Notably, the preferences of women diagnosed with cervical neoplasia regarding the delivery of bad news according to the SPIKES protocol have not been thoroughly evaluated. To address this gap, we used a survey based on the key elements of the SPIKES protocol. Our evaluation included three cohorts: women with cancer who underwent fertility-preserving surgery, those requiring radical hysterectomy or chemo-radiation, and women with cervical intraepithelial neoplasia grade 3 treated by loop excision. By gathering information on how bad news was communicated in reality and how patients envision an ideal communication process, our study aims to improve understanding and enhance the delivery of bad news in these specific clinical contexts.

## Methods

### Patients and recruitment procedure

Women diagnosed with cervical cancer at stage pT1A2 or pT1B1 with the option for fertility preservation underwent radical vaginal trachelectomy and laparoscopic lymph node dissection. This group is referred to as the RVT group, and out of 300 patients, 110 responded. The patients received treatment at the University of Jena, Charité University Berlin Campus Mitte and Campus Benjamin Franklin, or Asklepios Clinic Hamburg. Their written consent to be contacted for quality assessment and clinical research was obtained and documented digitally. The primary care gynecologists explained the diagnosis to the majority of patients, while discussions about therapy options took place with the gynecologic oncologists who performed the RVT.

Another group included women diagnosed with cervical cancer at FIGO stage I–III who were treated with nerve-sparing vaginal-assisted laparoscopic radical hysterectomy (VALRH) or laparoscopic lymphadenectomy staging followed by chemo-radiation. This group is referred to as the HE/RCT group, and out of 170 patients, 101 responded. All patients in this group were treated at the Clinics of the Institute for Cytology and Dysplasia (IZD) in Berlin, Dresden, or Chemnitz. Written consent for being contacted for quality assessment and clinical research was obtained and documented in their clinical files. Diagnosis was established histopathologically in the colposcopy clinic, and the colposcopists communicated the results to the patients who were then referred to a gynecologic oncologist (primarily C.K.) for further counseling and surgery.

The third group consisted of women diagnosed and treated for cervical intraepithelial neoplasia grade 3 (CIN 3) by loop excision and CO2 vaporization. This group is referred to as the CIN group, and out of 135 patients, 108 responded. All patients in this group were treated at the Clinics of the Institute for Cytology and Dysplasia (IZD) in Berlin. Written consent for being contacted for quality assessment and clinical research was obtained and documented in their clinical files. The breaking of bad news was performed by one physician (A.S.) who established the diagnosis and conducted the treatment.

All patients received a covering letter explaining the purpose of the study and asking for their cooperation by filling a questionnaire. The patients returned the questionnaire in a pre-paid envelope. The data were recorded anonymously in Excel.

### Measures

To assess the perceived and preferred breaking bad news quality, the Marburg Breaking Bad News Scale abbreviated MABBAN (von Blanckenburg et al. 2020) representing the six SPIKES subscales was used. These 38 items were delivered in a self-administered questionnaire (see Supplementary Appendix A) which is composed of 2 main parts: the first one asks for procedure, perception and satisfaction of the first cancer disclosure according to the recommended steps of the SPIKES protocol (e.g. my doctor took enough time; My doctor characterized the expected course of the disease in all clarity), while the second one consists of corresponding questions asking for the preference of patient’s assignment to these items (e.g., The doctor should take enough time; The doctor should characterize the expected course of disease in all clarity). The items were rated on a Likert scale from 1 (‘entirely’) to 4 (‘not at all’). Cronbachs Alpha was 0.93 for the reality items and *α* = 0.84 for the preference items. The catalogue of items applicable to patients diagnosed with CIN was shortened since some questions were not relevant for this group where disease has an excellent prognosis (see Supplementary Appendix B). Moreover, all patients had the possibility to give additional comments about important aspects of their BN experience in a short free text.

### Statistical analyzes

To provide descriptive statistics, we calculated the means, standard deviations, and percentages for all reality and preference variables. Next, we conducted the Wilcoxon signed-rank test to assess the significance of the differences between preferences and reality for each item. We specified the paired variables for the test, comparing the reality variables with the corresponding preference variables for each item. For the matched pairs Wilcoxon rank test, our prior recruitment goal was set to 208 participants (1–*ß* = 0.8, *α* = 0.05) to detect a small effect size calculated using G*Power (Faul et al. 2007). To analyze the differences among the three groups in terms of reality scores, we calculated an aggregate reality score based on the mean of all reality items. Subsequently, we performed a Kruskal–Wallis test, followed by a Bonferroni-corrected post hoc test for detailed group comparison. Moreover, the commentaries were rated by two independent raters as positive, negative or mixed.

## Results

### All patients

Whereas the age-range was similar between the three groups, patients in the RVT group were, due to the incentive to preserve fertility, younger than in the HE/RCT group and the CIN group (Table 2). When patients filled the questionnaire, time since diagnosis was brief for CIN patients since these women were treated only recently in 2021 or 2022. HE/RCT patients were treated between 2014 and 2022 and RVT patients between 1994 and 2022. This difference becomes even clearer by the percentage of patients which received their diagnosis within the last years with 85% in the CIN group, 16% in the HE/RCT group and only 4% in the RVT group.

**Table 2:** Patients’ profile

	RVT (*n* = 110)	HE/RCT (*n* = 101)	CIN (*n* = 108)
Age range (years)	21–47	36–73	26–78
Mean age (years), standard deviation	32.7 (4.73)	49.5 (11.80)	41.2 (10.38)
Mean time since diagnosis (months)	114	60	7
Diagnosis within the last year (%)	4	16	85

19 items with respect to patient’s preference compared to experienced reality are shown for all patients (Table 3): Preference ratings vary between 40 and 97% versus reality ratings between 24 and 86%. Patients’ preference higher than 90% was seen for four items which are contained in the SPIKES subscale 1 Setting: Ensure an undisturbed atmosphere (1), take enough time (1), explain the details of the disease comprehensible and in detail (1), give the patient enough possibilities to ask questions (1). The experienced reality for these four top items was significantly less satisfactory for patients varying between 73 and 86%.

**Table 3:** All patients

Item and number of SPIKES STEP	Patients’ preference		Reality		Test statistics		
	Entirely (%)	*M* (SD)	Entirely (%)	*M* (SD)	*z*	*r*	*n*
Ensure an undisturbed atmosphere STEP 1	92.1	1.09 (0.33)	73.0	1.36 (0.68)	− 6.02***	− 0.05 (n.s.)	313
Take enough time STEP 1	96.9	1.04 (0.26)	85.2	1.21 (0.56)	− 4.80***	0.03 (n.s.)	315
Explain the details of the disease comprehensible and in detail STEP 1	93.7	1.08 (0.32)	84.6	1.23 (0.62)	− 4.57***	0.24***	317
Give the patient enough possibilities to ask questions STEP 1	95.3	1.05 (0.26)	85.8	1.21 (0.58)	− 4.83***	0.31***	317
Reassure if the patient can understand everything STEP 1	88.4	1.13 (0.40)	78.6	1.30 (0.68)	− 4.53***	0.34***	314
Ask for the patient’s previous knowledge and what she further wants to know STEP 2	71.4	1.43 (0.75)	30.8	1.88 (1.04)	− 5.18***	0.24***	196
Ask about the patient’s concerns STEP 2	78.6	1.28 (0.60)	32.7	1.83 (1.04)	− 6.90***	0.44***	201
Announce the conversation STEP 3	44.7	1.47 (0.76)	44.3	1.50 (0.97)	− 0.43 (n.s.)	0.13 (n.s.)	200
Inform that he has to deliver bad news at the beginning of the talk STEP 3	39.9	1.66 (0.91)	29.9	1.96 (1.16)	− 3.58***	0.28***	190
Characterize the diagnosis in all clarity STEP 4	87.1	1.15 (0.46)	48.4	1.34 (0.67)	− 3.85***	0.31***	206
Characterize the expected course of disease in all clarity STEP 4	54.1	1.24 (0.53)	41.2	1.51 (0.79)	− 4.74***	0.31***	207
Try to be empathic STEP 5	83.0	1.20 (0.47)	77.7	1.29 (0.61)	− 2.40*	0.20***	314
Show interest in the patient’s feelings STEP 5	78.6	1.26 (0.56)	70.4	1.41 (0.73)	− 2.89**	0.26***	312
Show compassion STEP 5	64.5	1.50 (0.75)	40.3	1.52 (0.78)	− 1.07 (n.s.)	0.24***	204
Give the patient the possibility to show his/her feelings during the conversation STEP 5	54.7	1.22 (0.53)	40.9	1.55 (0.86)	− 5.38***	0.42***	201
Inform about effects of the disease on life circumstances STEP 6	56.3	1.18 (0.48)	38.1	1.69 (0.97)	− 6.82***	0.36***	202
Inform about possible therapies STEP 6	88.4	1.11 (0.43)	77.0	1.34 (0.74)	− 4.73***	0.07 (n.s.)	301
Inform about alternative treatment methods STEP 6	66.0	1.50 (0.84)	23.9	2.52 (1.36)	− 8.71***	0.42***	188
Involve the patient in further planning STEP 6	79.9	1.19 (0.47)	41.8	1.56 (0.90)	− 6.21***	0.18*	190

Highest ratings of patients’ preferences compared with experienced reality

*p < 0.05

**p < 0.01

***p < 0.001

Information defined by SPIKES subscale 2/3 (perception/invitation) such as Ask for the patient’s previous knowledge (2) and Ask about the patient’s concerns (2) was also significantly underrepresented and only offered to about one-third of patients whereas more than 70% of patients considered these items important.

SPIKES subscales 4 (knowledge), 5 (emotions) and 6 (summary/strategy) such as Give the patient the possibility to show his/her feelings during the conversation (5), Inform about effects of the tumor on life circumstances (6) Inform about alternative treatment methods (6) or Involve the patient in further planning (6) were only addressed in up to 42% of patients whereas up to more than 80% of women considered these items important when receiving BN.

### Group specific analyses

When we analyzed these results separately for each of the three groups results differ considerably: 58% in the RVT group, 71% in the HE RCT group, and 89% in the CIN group were entirely satisfied with the way they received BNs (see Supplementary Appendix C, D and E). The Kruskal–Wallis test yielded a significant result (*H* = 86.474, df = 2, *p* < 0.001), indicating significant differences among the groups in the aggregated reality variable. The Bonferroni corrected post hoc tests indicated significant differences between specific groups: RVT vs. CIN (*p* < 0.001), RVT vs. RCT (*p* = 0.036), and CIN vs. RCT (*p* < 0.001).

### RVT group

Women in the RVT group had been confronted with the diagnosis of a life-threatening disease leading to possible loss of fertility. Items such as Ensure an undisturbed atmosphere (1), Take enough time (1), Explain the details of the disease comprehensible and in detail (1), Give the patient enough possibilities to ask questions (1) and Inform about alternative treatment methods (6) are most important for these women but were significantly insufficiently addressed in the BBN process. For the majority of RVT patients, BN was broken by their gynecologist of choice and therapy was given by a gynecologic oncologist. In addition, the items Ask about the patient’s concerns (2), Give the patient the possibility to show his/her feelings during the conversation (5), Inform about effects of the tumour on life circumstances (6), Inform about alternative treatment methods (6) were significantly underrepresented in the BN presentation by the responsible gynecologist.

### HE/RCT group

For the patients in the HE/RCT group, six items representing five SPIKES sub scales reach at least 90% status in the preference ratings such as Ensure an undisturbed atmosphere (1), Take enough time (1), Explain the details of the disease comprehensible and in detail (1), Give the patient enough possibilities to ask questions (1), Reassure if the patient could understand everything (1), Inform about effects of the tumour on life circumstances (6). In addition, the five items Ask for the patient’s previous knowledge and what he further wants to know (2), Ask about the patient’s concerns (2), Characterize the expected course of disease in all clarity (4), Give the patient the possibility to show his/her feelings during the conversation (5), Inform about alternative treatment methods (6), were significantly underrepresented while BBN was performed by the responsible gynecologist.

### CIN group

Ensure an undisturbed atmosphere (1), Take enough time (1), Explain the details of the disease comprehensible and in detail (1) and Give the patient enough possibilities to ask questions (1) defined in SPIKES sub-scale 1 Setting were preferred by patients in more than 90% and satisfactorily experienced by the majority of women. With respect to significance show interest in the patient’s feelings (5) was overrepresented by 87 (reality) versus 79 percent (preference) according to patients answers.

### Personal comments

All patients had the possibility to give additional comments about important aspects of their BN experience (Table 4). From the RVT group, three quarters of patients gave an additional comment of which only 50% were exclusively positive, 38% were positive and negative and 12% only negative.

**Table 4:** Personal comments of patients

	RVT	HE/RCT	CIN
Comments made (%)	75	78	63
Positive (%)	50	95	82
Negative (%)	12	0	2
Mixed (%)	38	5	16

HE/RCT group has the highest rate of comments of 78% of which 95% were only positive and there was no negative comment in spite of the fact that treatment was drastic. From the CIN group gave two thirds an additional comment with only positive feedback by 82% of patients, 16% gave positive and negative comments and 2% only negative comments.

The following citations from patients’ comments illustrate problems which were frequently addressed:

“Patients should have the possibility to bring relatives to the conversation”.

“A second talk after a few days would have been valuable, since you do not realize many details. I still remember that the conversation took place in a dark room and it felt like heavy rain pouring down on me. I was 21 years old”.

“During the first talk I was shocked and could not capture all details. Questions arose only following the conversation after I came to turns with my diagnosis. Therefore I consider a second conversation after a few days as important”.

“There was a big conflict: to lie on a bench to get radiotherapy in order to save my life but at the same time to loose the possibility of bearing a child. Only following psycho-oncologic therapy I could get on the bench and tolerate radiation. My recommendation for other patients: psycho-oncologic support should be offered at the beginning”.

## Discussion

This study examined the practice of delivering bad news to German women diagnosed with cervical neoplasia. We investigated patients’ preferences compared to the perceptions of the actual delivery of bad news relating to the SPIKES-protocol. In total, many patients were satisfied with the communication process but in almost all SPIKES-categories the communication process of conveying challenging medical information to patients showed possibilities for improvement.

The dissonance between patient preferences and the reality of bad news delivery disjunction was not consistent across the treatment groups but extended to specific elements of the SPIKES protocol. The CIN group had almost no significant differences between the preference and reality scores showing higher levels of satisfaction. Thus, in the two other groups, most of the SPIKES scales setting, invitation/perception, emotions, knowledge, and strategy/summary showed discrepancies between the experiences patients made and the wishes they had. The most highly rated items belonged to the “Setting up” domain, including ensuring an undisturbed atmosphere and allowing enough time for the conversation. On the other hand, the most significant disparities were observed in the “Perception” and “Strategy/Summary” domains. This is in line with previous findings of bad news delivery in a large sample of cancer patients (Seifart et al. 2014), showing that numerous important communication aspects were not fulfilled.

The most notable disparities between patients’ perceived reality and their preferences were evident in four specific communication aspects: for instance, the discrepancy seen in “involve the patient in further planning” highlights the need for healthcare providers to actively engage patients in the decision-making process. Only 42% of patients reported experiencing involvement in further planning, while 80% expressed a preference for such engagement. This emphasizes a communication gap that could potentially impact patients’ feelings of control and satisfaction with their care journey. Moreover, the finding is in line with the results of Polish researchers who found in a sample of 226 cancer patients that the SPIKES domains setting up, knowledge and emotions were delivered in a satisfying way, but perception, invitation and strategy and summary need improvement by more training (Marschollek et al. 2019).

“Inform about effects of the disease on life circumstances” also showed a discrepancy, with 56% of patients preferring to receive this information compared to the lower 48% who reported it in reality. This gap signifies an opportunity for clinicians to better address the holistic impact of the disease on patients’ lives, fostering a more comprehensive and patient-centered approach to communication. Moreover, fertility concerns may play a role here. Empathy discrepancies raises concerns about the potential emotional toll faced by patients who perceive their hopes being hindered. This resonates with previous research highlighting the significance of acknowledging patients’ hopes and aspirations during bad news delivery (Baile et al. 2000). Incorporating frameworks like the HOPES acronym can facilitate a more holistic approach that factors in patients’ emotional well-being and their hopes for the future (Temple 2018; Whitney et al. 2008).

A similar pattern was found in the item “inform about alternative treatment methods” that was part of the conversation in 24% of the patients but wished by 66%. Interestingly, our study’s outcomes reveal a disparity in the delivery of empathetic asking during the conversation. The contrast between “ask about the patient’s concerns” in reality (33%) and patient preference (79%) emphasizes the significance of actively seeking and addressing patients’ concerns during discussions. Furthermore, the gap observed in the item “Ask for the patient’s previous knowledge and what she further wants to know” could indicate that patients should be granted a more proactive role in the conversation. Moreover, as Seifart et al. (2014) recommend a two-step process, a second conversation could be useful to address concerns that patients may have forgotten during the initial shock of diagnosis transmission (Mirza et al. 2019). Helpful may be a short pre-appointment communication, that encourages patients to submit questions or concerns in advance of the BBN conversation.

An important issue, that could have a great influence on the quality of breaking bad news could be the time component. Time is a precious commodity in nowadays health care systems and BBN can easily take a considerable amount of time, the more so when a second or possible third follow-up meeting is considered. Thus, some considerations for an effective time management can be: providing educational materials (Mirza et al. 2019) and resources in advance of BBN encounters; forming interdisciplinary teams that include not only oncologists and gynecologists, but also family physicians, social workers (Spiegel et al. 2009), and nurses can distribute the communication workload (Wan et al. 2020); utilizing telemedicine and virtual communication platforms that can facilitate ongoing communication between patients and healthcare providers; providing healthcare professionals with training in time management and effective communication techniques can help them convey information efficiently without compromising empathy and compassion; and patient-centered care planning: collaboratively developing care plans with patients and their families can help set clear expectations and prioritize the most critical topics for discussion during BBN encounters, reducing the time required for decision-making.

Our study’s findings also align with international literature suggesting opportunities for improvement in breaking bad news procedures (Fallowfield and Jenkins 2004). Several countries have explored innovative approaches to communication skills training, such as Switzerland’s use of simulated patients (Carrard et al. 2020) and the Netherlands’ emphasis on diverse feedback sources (Brouwers et al. 2019). In a continuous feedback loop, a feedback mechanism where patients and families can provide input on their BBN experience could be established. This feedback can inform ongoing improvements in communication practices. A French virtual peer role-play model, introduced in response to the pandemic, highlights the potential of technology in training effective communication (Bouaoud et al. 2022). In a study from Belgium, it could be demonstrates that training triadic communication skills improves the communication skills and should be included in resident curriculum (Merckaert et al. 2013).

The COVID-19 pandemic has further catalyzed changes in communication dynamics, necessitating adaptability in the way bad news is conveyed (Hauk et al. 2021). The shift to remote communication methods introduced a new layer of challenges that warrant exploration in future research. The study by Goumas et al. (2023) underscores the need for studies assessing the impact of distance communication on both patients and healthcare professionals in the era of pandemic-driven virtual interactions. Such research can inform the adaptation of communication practices to evolving healthcare landscapes.

This study has several potential limitations. Patients were asked to recall their initial diagnosis disclosure, and it is likely that their perception and emotional state changed over time. The mean interval to diagnosis was 1/2 year for the CIN group, 10 years for the RVT group, and 5 years for the HE/RCT group which could have led to recall bias and has to be considered in the interpretation of the data. Additionally, the return rates of questionnaires differed considerably among the three groups (70% for CIN, 35% for RVT, and 20% for HE/RCT). This fact could also produce bias because maybe only the persons especially satisfied or dissatisfied answered to the study request. This could impact the generalizability of the findings to the broader population of individuals with the respective conditions. Nevertheless, even if the return rates differ, the data from those who responded provide valuable information relevant to understand the specific group and their experiences. Another limitation is the lack of information about physicians’ communication approaches, styles, training in communication skills, and preferences for breaking bad news in a cross-cultural context. The survey and questionnaire were based on the SPIKES protocol’s recommendations, potentially missing other important aspects. Furthermore, the questionnaires were distributed and answered during the COVID-19 pandemic, a stressful period that could have influenced patients’ emotional states and their responses.

Future research could consider a longitudinal approach that accounts for patients’ evolving perceptions over time. Additionally, it could be important to investigate physicians’ cross-cultural communication competencies**.** Furthermore, integrating patient feedback mechanisms within oncology institutions can serve as a valuable tool for quality control, ensuring continuous improvement in bad news delivery practices.

In conclusion, our study emphasizes the need for improved bad news delivery practices in the realm of cervical neoplasia diagnoses. By acknowledging the communication discrepancies between patient preferences and the perceived reality, we can shape a more empathetic and patient-centered approach that respects individual differences and hopes and may reassure a more proactive role of the patient. As most relevant clinical implications, it can be recommended taking a second conversation a few days later as a two-step approach, including relatives into the communication process, granting patients a more proactive role in the conversation and including the patients in further treatment planning. As healthcare landscapes continue to evolve, integrating innovative communication training methods will be essential in bridging the gap between patients’ emotional needs and the communication process.

## Supplementary Information

Below is the link to the electronic supplementary material.

Supplementary file1 (DOCX 1290 KB)

## Data Availability

The data are available from the corresponding author, PvB, upon reasonable request.
