# Supplementary material for: Quality of breaking bad news to patients diagnosed with neoplasia of the uterine cervix
Source: J Cancer Res Clin Oncol. 2023 Oct 4;149(19):17215–22. doi: 10.1007/s00432-023-05442-2 (PMC10657321; doi:10.1007/s00432-023-05442-2)
Supplement: Supplementary file 1 — Supplementary file1 (DOCX 1290 KB) [file 432_2023_5442_MOESM1_ESM.docx]

### Appendix A

### Mabban questionnaire
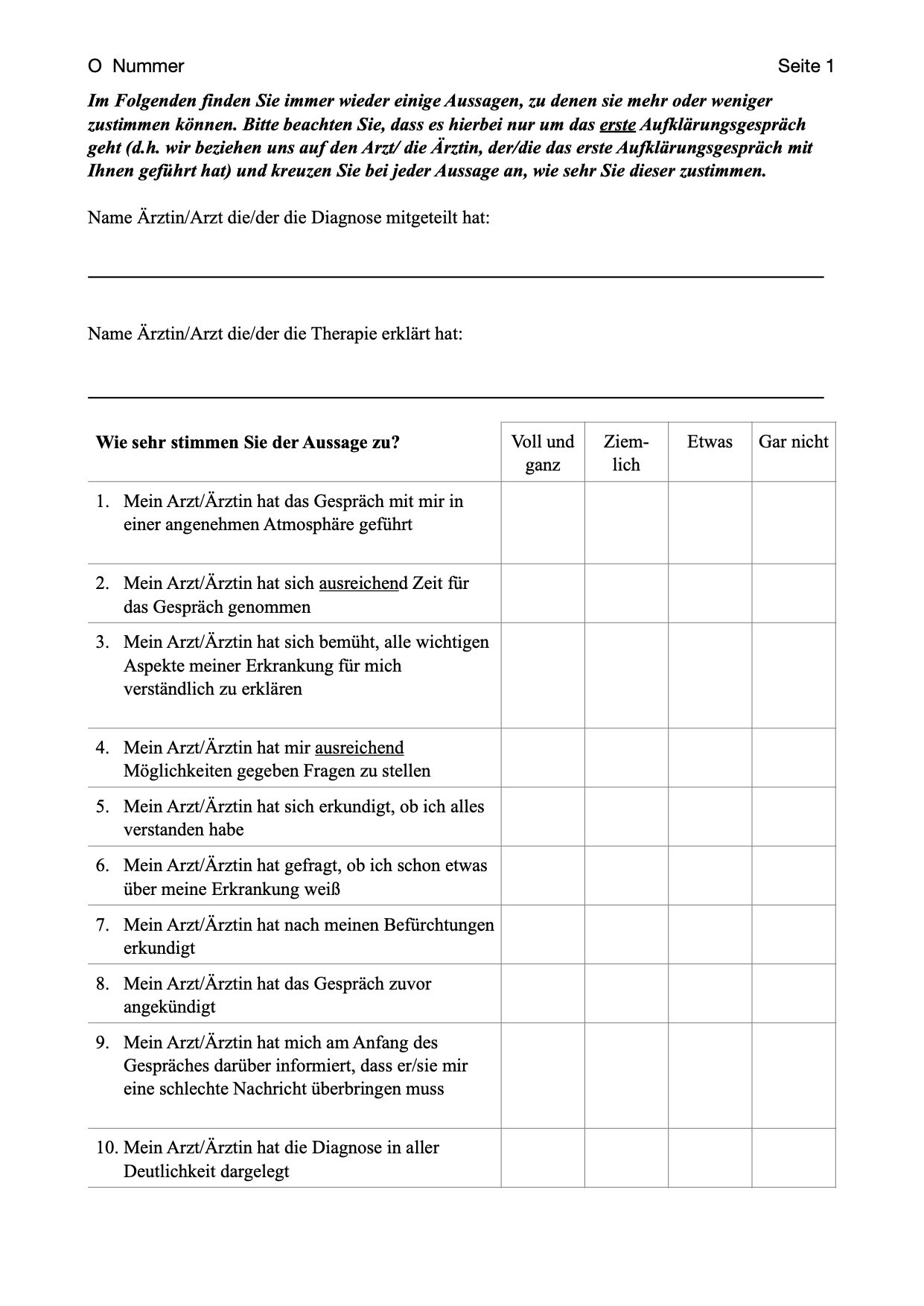
 patients RVT and HE/RCT group


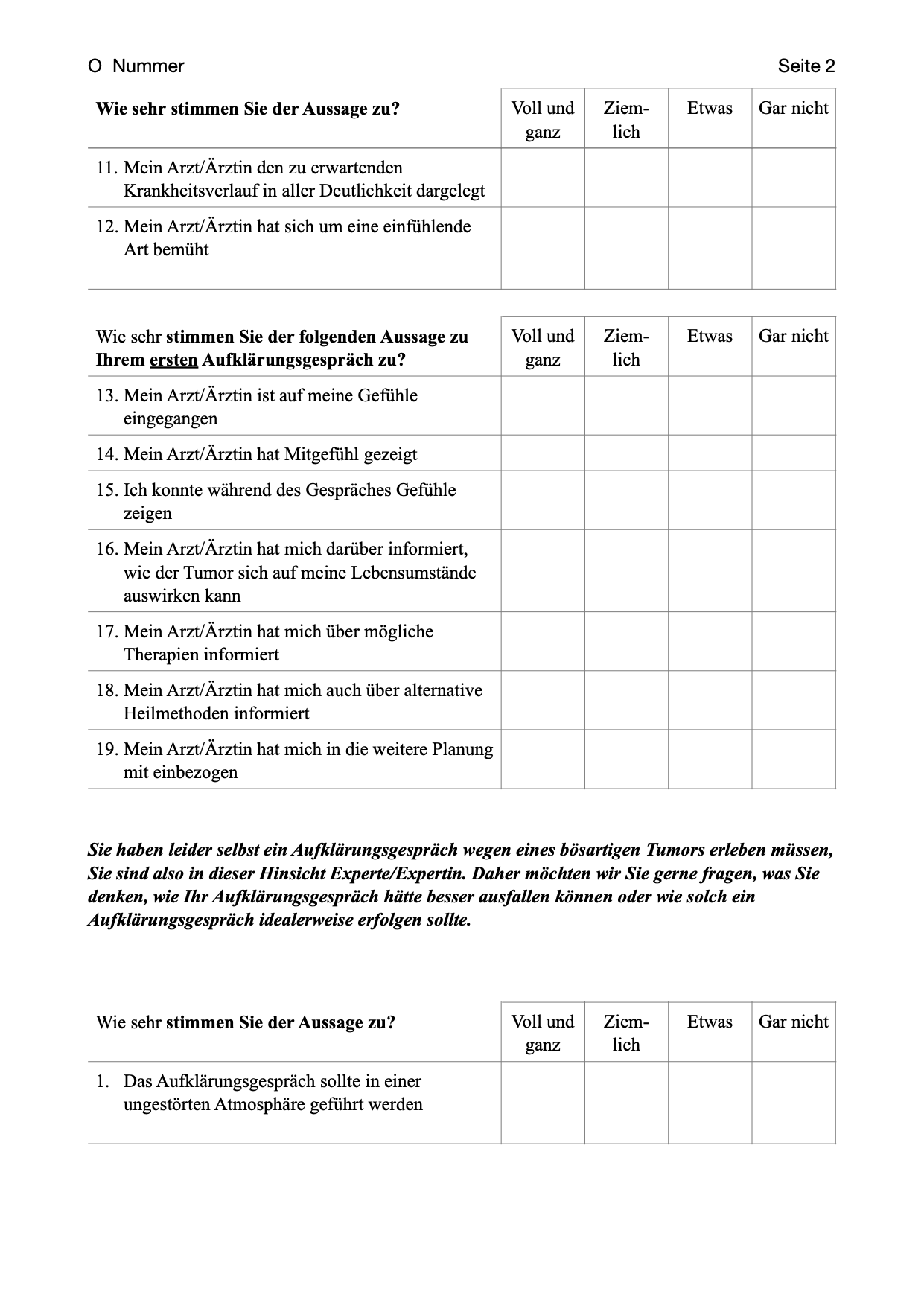


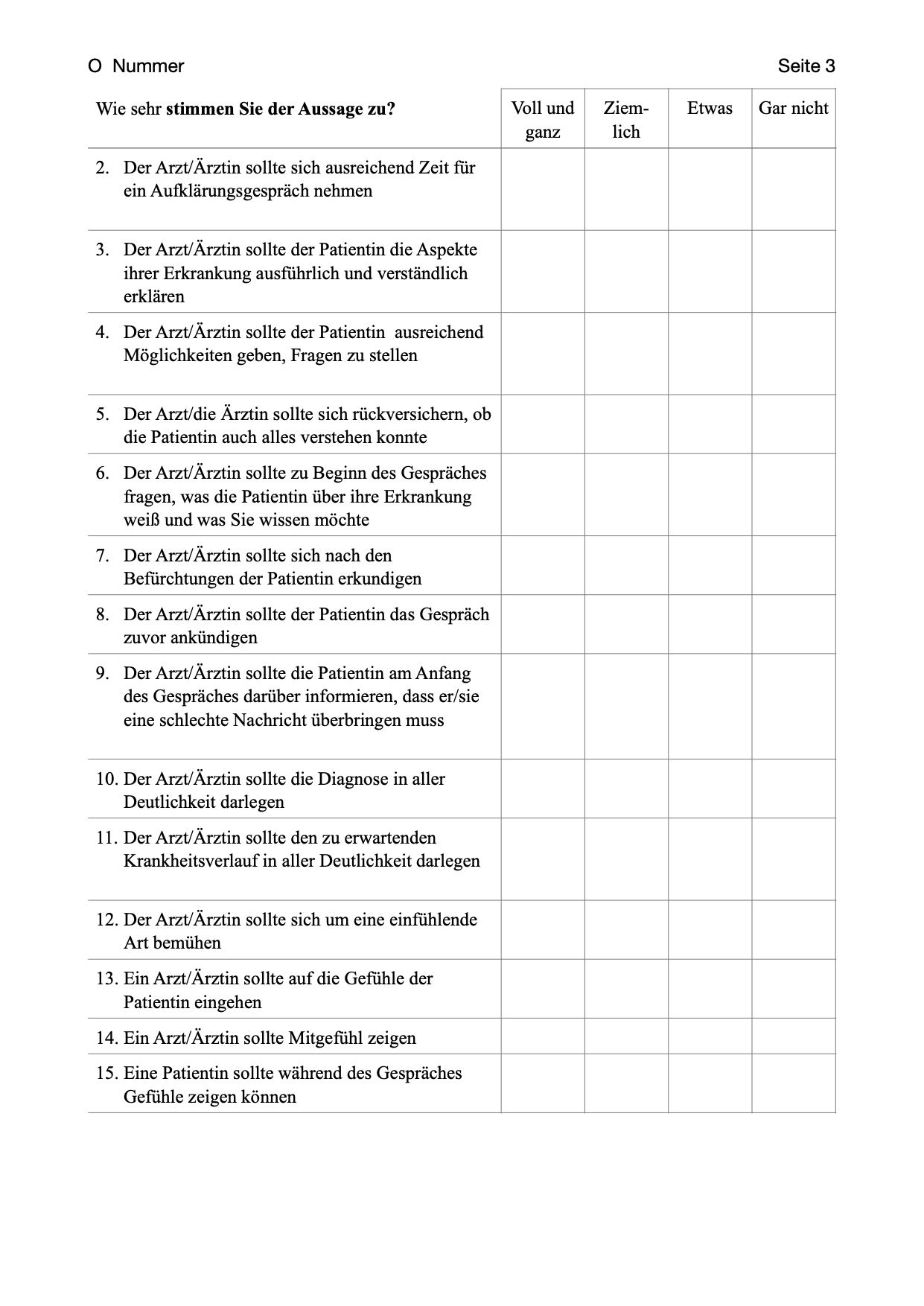


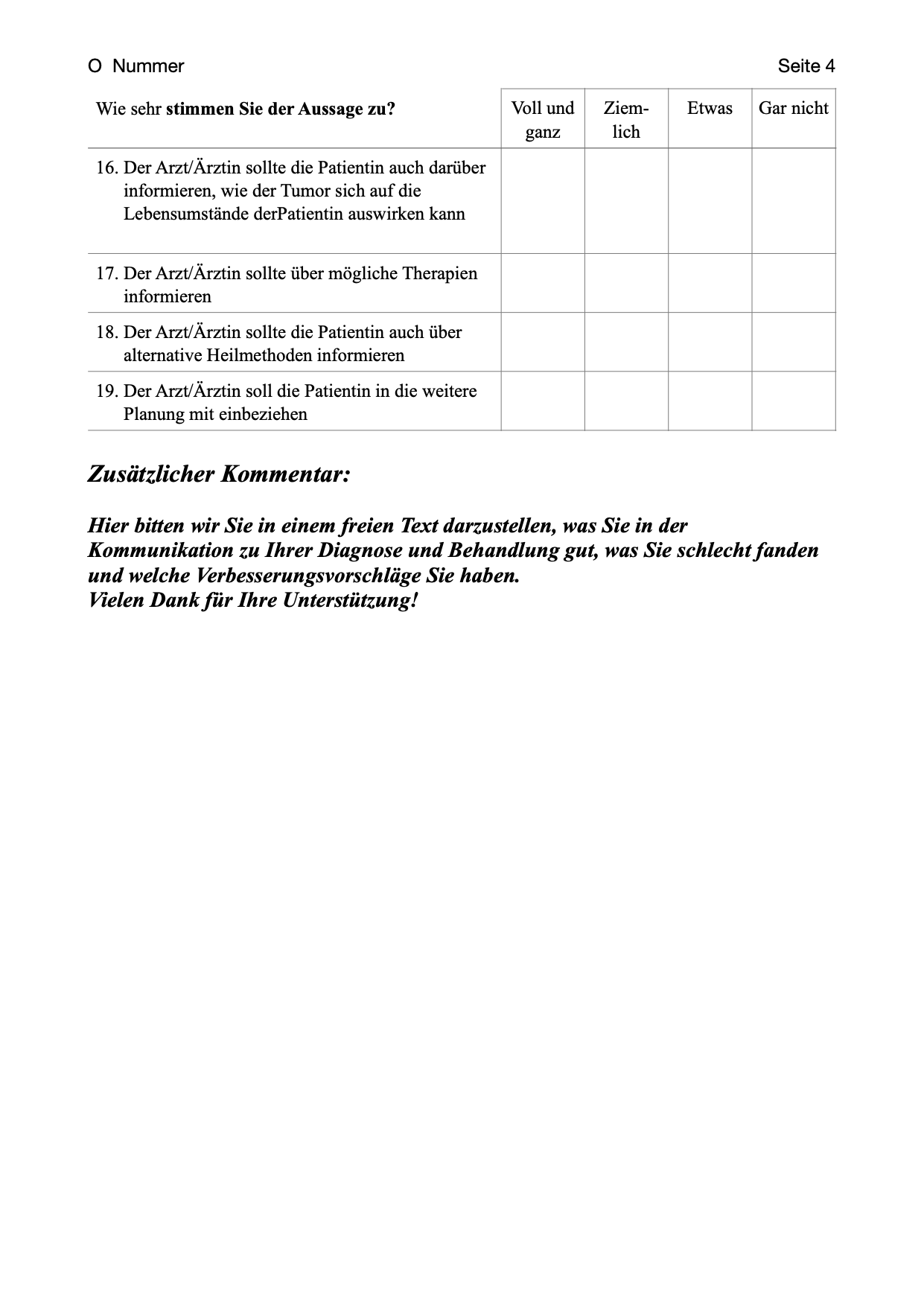


### Appendix B

### Mabban question
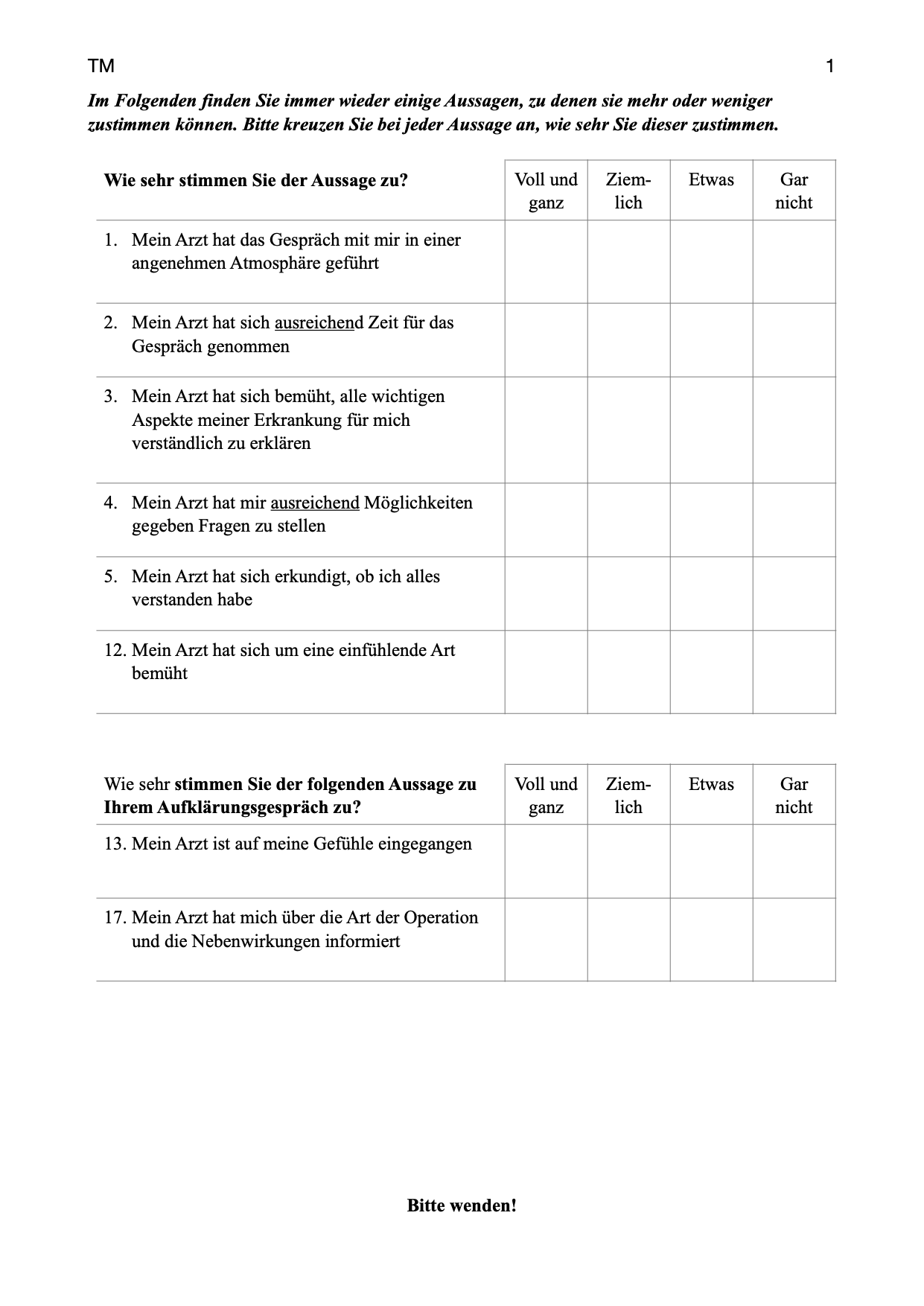
naire patients CIN group


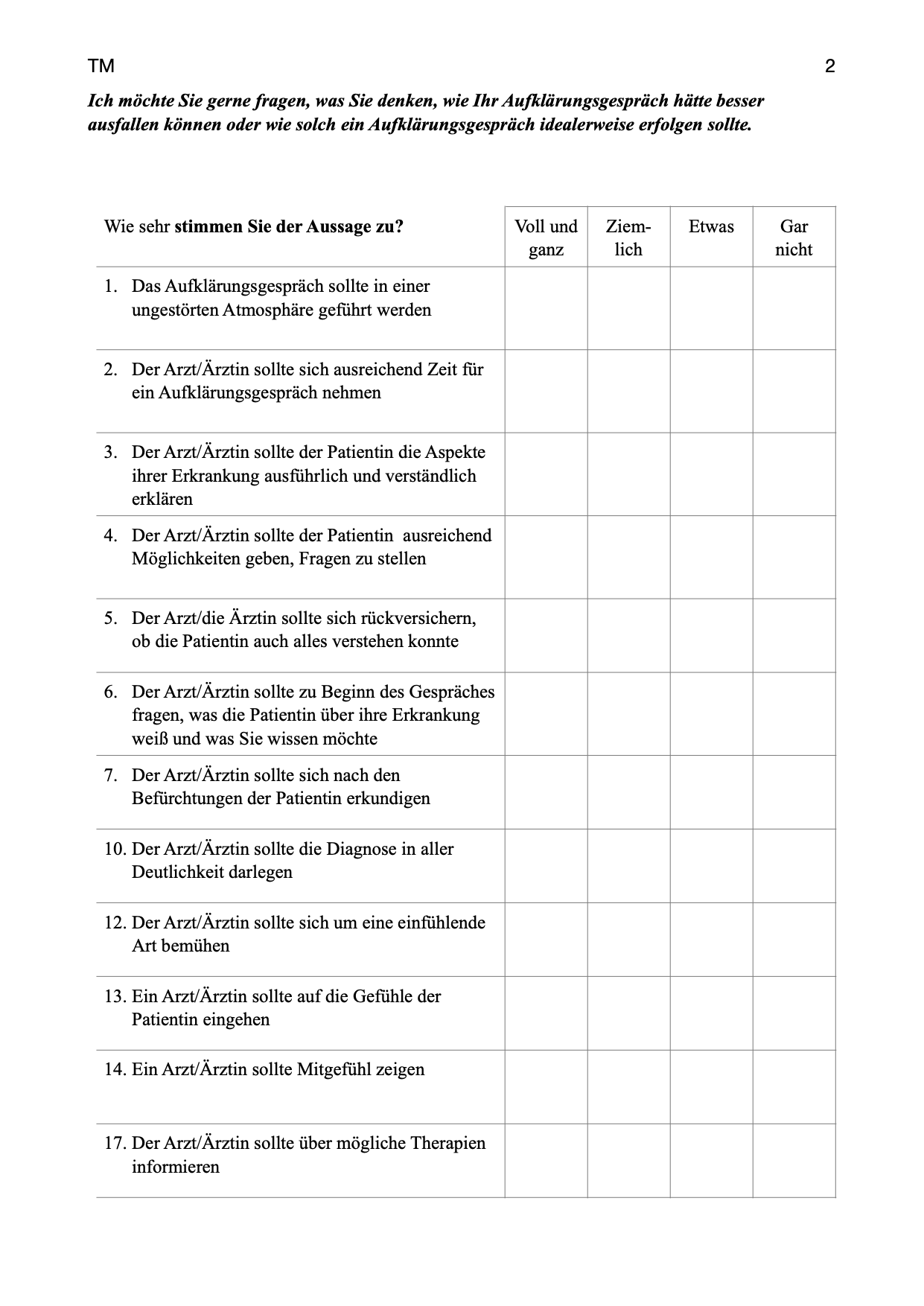


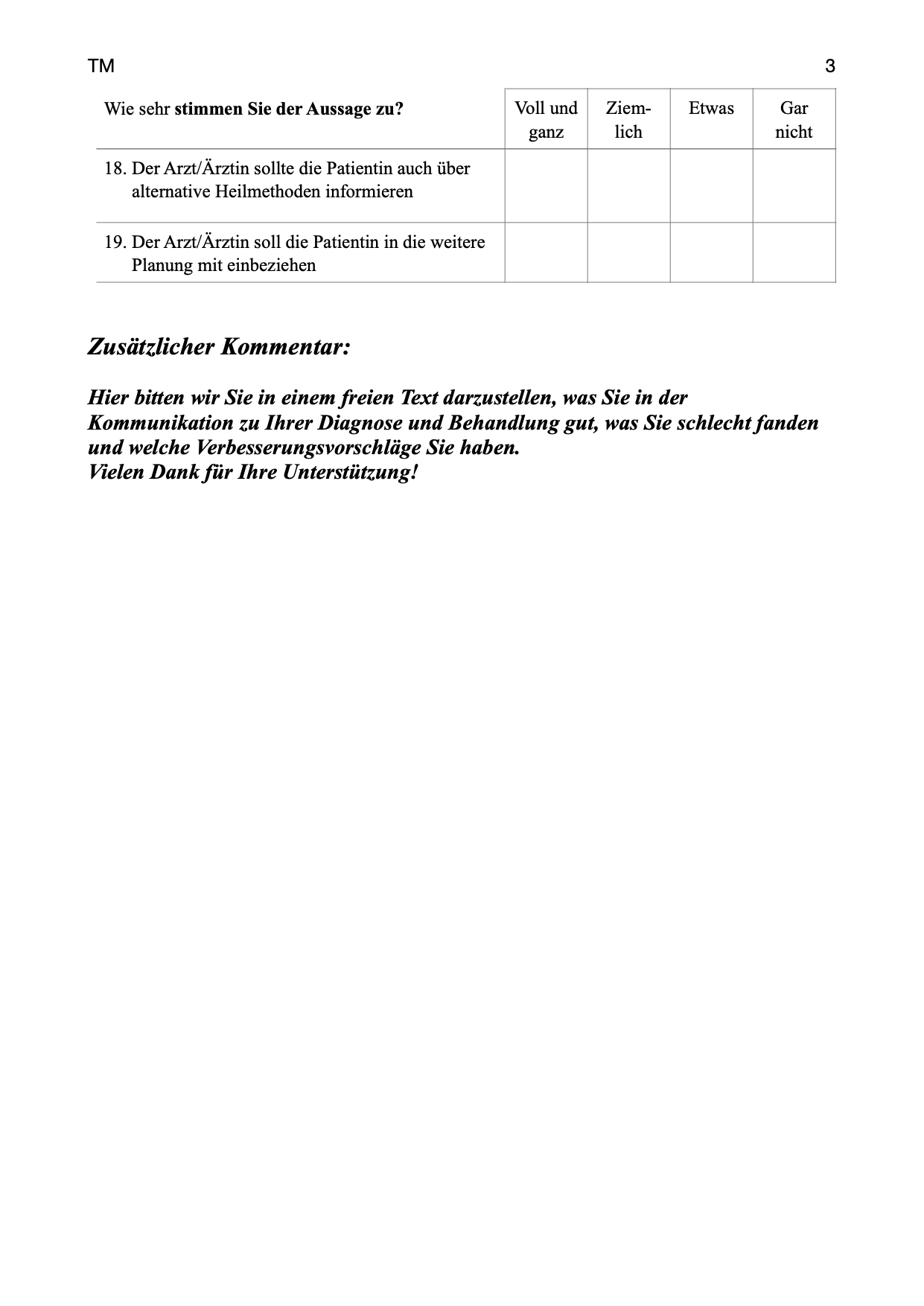


### Appendix C

### Group RVT – Cervical cancer treated by Radical vaginal trachelectomy (RVT). Highest ratings of patients preferences compared with experienced reality

| **Item and # of SPIKES sub scale** | **Patients‘ preference** | | **Reality** | | **Test statistics** | | |
| --- | --- | --- | --- | --- | --- | --- | --- |
|  | **‚Entirely‘ (%)** | **M (SD)** | **‚Entirely‘ (%)** | **M (SD)** | **z** | **r** | **n** |
| **Ensure an undisturbed atmosphere 1** | 94.5 | 1.05 (0.25) | 53.6 | 1.61 (0.81) | -5.74*** | -0.05 (n.s.) | 106 |
| **Take enough time 1** | 95.5 | 1.04 (0.23) | 80.9 | 1.25 (0.56) | -3.32*** | -0.07 (n.s.) | 108 |
| **Explain the details of the disease comprehensible and in detail 1** | 90.0 | 1.10 (0.30) | 82.7 | 1.25 (0.59) | -2.46* | 0.17 (n.s.) | 110 |
| **Give the patient enough possibilities to ask questions 1** | 93.6 | 1.06 (0.25) | 81.8 | 1.23 (0.54) | -3.26** | 0.38*** | 110 |
| **Reassure if the patient could understand everything 1** | 87.3 | 1.13 (0.36) | 77.3 | 1.31 (0.68) | -2.92** | 0.44*** | 107 |
| **Ask for the patient’s previous knowledge and what he further wants to know 2** | 62.7 | 1.59 (0.85) | 43.6 | 1.97 (1.07) | -3.20** | 0.37*** | 103 |
| **Ask about the patient’s concerns 3** | 73.6 | 1.35 (0.66) | 40.9 | 2.01 (1.10) | -5.57*** | 0.51*** | 102 |
| **Announce the conversation 3** | 59.1 | 1.62 (0.85) | 60.0 | 1.69 (1.04) | -0.66 (n.s.) | 0.09 (n.s.) | 103 |
| **Inform that he has to deliver bad news at the beginning of the talk 3** | 50.0 | 1.86 (0.97) | 30.9 | 2.18 (1.16) | -2.47* | 0.28** | 90 |
| **Characterize the diagnosis in all clarity 4** | 85.5 | 1.17 (0.45) | 71.8 | 1.36 (0.68) | -2.65** | 0.33*** | 107 |
| **Explanation of course of disease 6** | 77.3 | 1.29 (0.58) | 59.1 | 1.53 (0.77) | -3.09** | 0.35*** | 107 |
| **Try to be empathic 5** | 84.5 | 1.19 (0.48) | 64.5 | 1.48 (0.76) | -3.25** | 0.16 (n.s.) | 108 |
| **Show interest in the patient’s feelings 5** | 76.4 | 1.29 (0.57) | 55.5 | 1.61 (0.81) | -3.47*** | 0.21* | 108 |
| **Show compassion** **5** | 60.9 | 1.54 (0.76) | 49.1 | 1.67 (0.77) | -1.44 (n.s.) | 0.28** | 107 |
| **Give the patient the possibility to show his/her feelings during the conversation 5** | 80.9 | 1.23 (0.52) | 55.5 | 1.64 (0.88) | -4.61*** | 0.46*** | 104 |
| **Inform about effects of the disease on life circumstances 6** | 79.1 | 1.23 (0.52) | 54.5 | 1.75 (1.00) | -4.84*** | 0.42*** | 104 |
| **Inform about possible therapies 6** | 92.7 | 1.07 (0.32) | 60.0 | 1.61 (0.93) | -4.65*** | 0.01 (n.s.) | 104 |
| **Inform about alternative treatment methods 6** | 61.8 | 1.54 (0.82) | 23.6 | 2.81 (1.29) | -6.93*** | 0.43*** | 97 |
| **Involve the patient in further planning 6** | 85.5 | 1.16 (0.46) | 52.7 | 1.76 (1.00) | -5.26*** | 0.17 (n.s.) | 104 |

* p < 0.05 ** p < 0.01 *** p < 0.001

### Appendix D

### Group HE-RCT – Cervical cancer treated by radical hysterectomy (HE) or radiochemotherapy (RCT). Highest ratings of patients preferences compared with experienced reality

| **Item and # of SPIKES sub scale** | **Patients‘ preference** | | **Reality** | | **Test statistics** | | |
| --- | --- | --- | --- | --- | --- | --- | --- |
|  | **‚Entirely‘ (%)** | **M (SD)** | **‚Entirely‘ (%)** | **M (SD)** | **z** | **r** | **n** |
| **Ensure an undisturbed atmosphere 1** | 90.0 | 1.10 (0.30) | 78.8 | 1.32 (0.71) | -2.86** | 0.18 (n.s.) | 99 |
| **Take enough time 1** | 98.0 | 1.03 (0.22) | 84.8 | 1.23 (0.64) | -3.09** | 0.24* | 99 |
| **Explain the details of the disease comprehensible and in detail 1** | 91.9 | 1.10 (0.36) | 77.8 | 1.35 (0.76) | -3.46*** | 0.42*** | 99 |
| **Give the patient enough possibilities to ask questions 1** | 93.9 | 1.06 (0.24) | 80.8 | 1.32 (0.77) | -3.68*** | 0.57*** | 99 |
| **Reassure if the patient could understand everything 1** | 90.0 | 1.13 (0.42) | 76.8 | 1.36 (0.76) | -3.15** | 0.38*** | 99 |
| **Ask for the patient’s previous knowledge and what he further wants to know 2** | 82.8 | 1.23 (0.57) | 50.5 | 1.75 (0.99) | -3.99*** | -0.02 (n.s.) | 93 |
| **Ask about the patient’s concerns 3** | 87.9 | 1.19 (0.57) | 58.6 | 1.64 (0.98) | -4.14*** | 0.31** | 99 |
| **Announce the conversation 3** | 74.7 | 1.32 (0.62) | 74.7 | 1.30 (0.84) | -0.16 (n.s.) | 0.08 (n.s.) | 97 |
| **Inform that he has to deliver bad news at the beginning of the talk 3** | 69.7 | 1.45 (0.80) | 60.6 | 1.77 (1.14) | -2.26* | 0.22* | 100 |
| **Characterize the diagnosis in all clarity 4** | 88.9 | 1.13 (0.42) | 74.7 | 1.34 (0.67) | -2.81** | 0.28** | 99 |
| **Explanation of course of disease 6** | 84.8 | 1.18 (0.46) | 65.7 | 1.49 (0.81) | -3.64*** | 0.28* | 100 |
| **Try to be empathic 5** | 83.8 | 1.20 (0.49) | 81.8 | 1.26 (0.62) | -0.84 (n.s.) | 0.19 (n.s.) | 100 |
| **Show interest in the patient’s feelings 5** | 80.8 | 1.26 (0.60) | 68.7 | 1.46 (0.83) | -2.02* | 0.18 (n.s.) | 98 |
| **Show compassion 5** | 73.7 | 1.36 (0.68) | 73.7 | 1.36 (0.76) | -0.03 (n.s.) | 0.14 (n.s.) | 97 |
| **Give the patient the possibility to show his/her feelings during the conversation 5** | 83.8 | 1.21 (0.54) | 69.7 | 1.45 (0.82) | -2.78** | 0.39*** | 97 |
| **Inform about effects of the disease on life circumstances 6** | 90.9 | 1.11 (0.40) | 60.6 | 1.63 (0.95) | -4.83*** | 0.27** | 98 |
| **Inform about possible therapies 6** | 85.9 | 1.07 (0.33) | 81.8 | 1.27 (0.65) | -3.00** | 0.21* | 90 |
| **Inform about alternative treatment methods 6** | 74.7 | 1.36 (0.79) | 49.5 | 2.23 (1.37) | -5.39*** | 0.38*** | 92 |
| **Involve the patient in further planning 6** | 79.8 | 1.10 (0.31) | 74.7 | 1.36 (0.74) | -3.24** | 0.18 (n.s.) | 87 |

* p < 0.05 ** p < 0.01 *** p < 0.001

### Appendix E

### Group CIN – Cervical intraepithelial neoplasia grade 3 treated by loop excision(CIN 3). Highest ratings of patients preferences compared with experienced reality

| **Item and # of SPIKES sub scale** | **Patients‘ preference** | | **Reality** | | **Test statistics** | | |
| --- | --- | --- | --- | --- | --- | --- | --- |
|  | **‚Entirely‘ (%)** | **M (SD)** | **‚Entirely‘ (%)** | **M (SD)** | **z** | **r** | **n** |
| **Undisturbed atmosphere 1** | 91.7 | 1.11 (0.42) | 87.0 | 1.16 (0.46) | -0.84 (n.s.) | -0.04 (n.s.) | 108 |
| **Take enough time 1** | 97.2 | 1.05 (0.32) | 89.8 | 1.14 (0.46) | -1.84 (n.s.) | -0.04 (n.s.) | 108 |
| **Explain the details of the disease comprehensible and in detail 1** | 99.1 | 1.03 (0.29) | 92.6 | 1.12 (0.47) | -1.68 (n.s.) | -0.03 (n.s.) | 108 |
| **Give the patient enough possibilities to ask questions 1** | 98.1 | 1.04 (0.30) | 94.4 | 1.08 (0.37) | -1.07 (n.s.) | -0.03 (n.s.) | 108 |
| **Reassure if the patient could understand everything 1** | 88.0 | 1.14 (0.42) | 82.4 | 1.21 (0.53) | -1.47 (n.s.) | 0.24* | 108 |
| **Try to be empathic 5** | 80.6 | 1.20 (0.45) | 87.0 | 1.13 (0.34) | -1.61 (n.s.) | 0.45*** | 106 |
| **Show interest in the patient’s feelings 5** | 78.7 | 1.24 (0.51) | 87.0 | 1.15 (0.43) | -2.30* | 0.54*** | 106 |
| **Informed about possible therapies 6** | 87.0 | 1.19 (0.57) | 89.8 | 1.16 (0.52) | -0.19 (n.s.) | 0-16 (n.s.) | 108 |

- p < 0.05 ** p < 0.01 *** p < 0.001
